# Intriguing Electrostatic Potential of CO: Negative Bond-ends and Positive Bond-cylindrical-surface

**DOI:** 10.1038/srep16307

**Published:** 2015-11-06

**Authors:** Hahn Kim, Van Dung Doan, Woo Jong Cho, Rosendo Valero, Zahra Aliakbar Tehrani, Jenica Marie L. Madridejos, Kwang S. Kim

**Affiliations:** 1 Department of Chemistry, Korea Advanced Institute of Science and Technology, Daejeon 305-701, Korea; 2 Department of Chemistry, School of Natural Science, Ulsan National Institute of Science and Technology (UNIST), Ulsan 689-798, Korea

## Abstract

The strong electronegativity of O dictates that the ground state of singlet CO has positively charged C and negatively charged O, in agreement with ab initio charge analysis, but in disagreement with the dipole direction. Though this unusual phenomenon has been fairly studied, the study of electrostatic potential (EP) for noncovalent interactions of CO is essential for better understanding. Here we illustrate that both C and O atom-ends show negative EP (where the C end gives more negative EP), favoring positively charged species, whereas the cylindrical surface of the CO bond shows positive EP, favoring negatively charged ones. This is demonstrated from the interactions of CO with Na^+^, Cl^–^, H_2_O, CO and benzene. It can be explained by the quadrupole driven electrostatic nature of CO (like N_2_) with very weak dipole moment. The EP is properly described by the tripole model taking into account the electrostatic multipole moments, which has a large negative charge at a certain distance protruded from C, a large positive charge on C, and a small negative charge on O. We also discuss the EP of the first excited triplet CO.

Carbon monoxide, though toxic, is an important species present in our environment and biosystems as it is one of the most abundant molecules. Since CO is widely used as a ligand and a reducing agent in chemistry, including C_1_ chemistry as well as mineral industries, it is of importance to correctly understand interactions of CO with various molecules. Non-covalent interactions^1^,^2^,^3^ govern molecular recognition and molecular organization/assembly, depending on magnitudes and differences in interaction energy components such as electrostatic interaction, induction, dispersion and exchange repulsion. Oftentimes, strong binding arises from electrostatic interactions. This type of interaction is usually understood based on atomic charges in each molecule. Each atomic charge is generally described in terms of isotropic point charge. However, the EP of CO cannot be simply described by isotropic point charges of C and O. Despite that covalent interactions involving with CO have been fairly studied, the study of EP for CO is essential for better understanding of noncovalent interactions of CO as well as for removal of confusion and misunderstanding of interactions involved with CO.

The ground state of CO is a singlet state with a triple bond comprised of two covalent bonds and one dative covalent bond. The bond dissociation energy of CO (1072 kJ/mol) is the strongest chemical bond, stronger than the N_2_ triple bond (942 kJ/mol)^4^. The oxidation state of C in CO is +2. According to the octet rule, four of the shared electrons in C come from O and only two come from C, so one bonding orbital is occupied by two electrons from O, forming a dative or dipolar bond. This causes a C ← O polarization of the molecule. Thus, it could be considered that a small negative charge is formed on C and a small positive charge on O. Even though two bonding orbitals (each occupied by one electron from C and one from O) form slightly polar covalent bonds to reverse the C → O polarization (as O is more electronegative than C), the dative bonding orbital results in a negative net charge δ^−^ at the C end which gives a small dipole moment (0.11 D)^5^,^6^,^7^,^8^,^9^ pointing from C to O.

On the other hand, the quantum theory of atoms in molecules analysis showed that the C/O is positively/negatively charged, respectively. This is consistent with the natural bond orbital (NBO) charge of C/O which is +0.44/−0.44 au at the level of Moller-Plesset second order perturbation (MP2) theory using the aVTZ basis set (where aVNZ denotes aug-cc-pVNZ; N = D/T/Q/5). Since CO has a small dipole moment with the negative end at the C atom, it is argued that the apparent anomaly for CO arises from the severe polarization of electron density on the C atom overriding the effect of electronegativity difference^10^,^11^,^12^. If only the dipole direction of CO is simply considered, one could erroneously expect the charge distribution of negatively/positively charged C/O.

In this regard, we investigated the anisotropic charge distribution of CO. It shows an intriguing EP map for the singlet ground state of CO (Fig. 1a). Both EPs of the C and O atom-ends along the CO bond axis (z axis) are negative, with the C atom-end being more negative. In contrast, EP of the cylindrical surface of the CO bond between C and O is positive.

## Results

**Figure 1:**
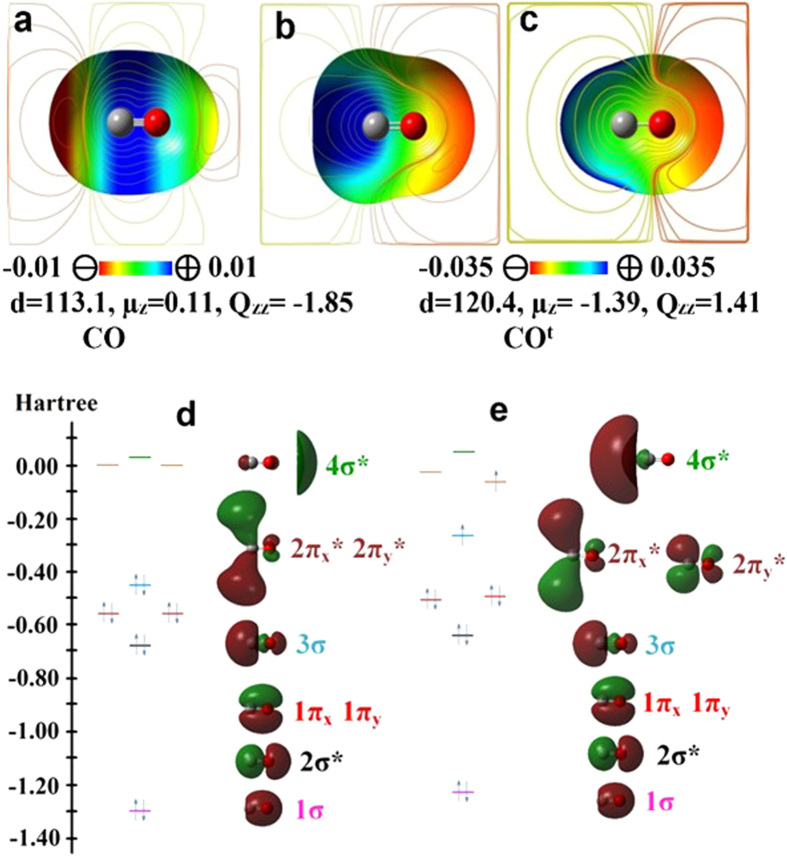
EP maps, electronic properties and frontier MOs of the ground state CO (X ^1^Σ^+^) and the first excited triplet state CO^t^ (a ^3^Π). EP maps for the ground (**a**) and first excited ((**b**) top and (**c**) front views) states of CO at the MP2/aVTZ level (density isovalue: 0.001 au). Bond-length (*d* in pm), dipole moment (*μ*_z_ in D), and quadrupole moment (*Q*_zz_ in D^+^Å) are given at the CCSD(T)/aV5Z level. For the singlet CO, both C and O atom-ends show negative EP, whereas the cylindrical surface of the CO bond, positive EP. For CO^t^, the C atom-end gives positive EP, whereas the O atom-end, negative EP. MO energies and orbital shapes for (**d**) CO and (**e**) CO^t^ are given at the M06-2X/aVTZ level.

Simple molecular orbital (MO) picture based on atomic orbital overlaps have difficulties in providing a proper explanation for the EP of CO. The two π bonding MOs and one σ bonding MO in CO form a triple bond (Fig. 1d). For all the bonding MOs, regardless of σ or π bonds, the electron density increases between the two atoms due to the orbital overlap, but decreases outside the two atoms. One might be tempted to think that the cylindrical surface would have negative EP, while the atom-ends would have positive EP, as opposed to the EP map in Fig. 1a.

The high electronegativity of O tends to keep significant electron population around the O atom-end. In the proximity of the C/O nuclei, the positive EP due to the C/O nuclei is not screened out by the electron density. Far from the nuclei, the positive EP due to the nuclei is over-screened, resulting in negative EP around the C and O atom-ends. Nevertheless, a large amount of electron population is between C and O because of the triple bond nature of CO. The CO triple bond length (113 pm) is very short, comparable to the N_2_ triple bond length (110 pm), much shorter than the O_2_ double-bond length (121 pm)^5^. Therefore, the six electrons cannot be accommodated within the short CO bond between C and O nuclei; their significant electron population is outside the C and O nuclei, resulting in negative EP in both atom-end regions and positive EP in the region between the C and O atoms. Since C has the smaller effective nuclear charge than O ((Z_eff_(C_2p_) = 3.14 vs. Z_eff_(O_2p_) = 4.45)^13^, the C atom-end has the weaker nucleus-charge screening effect. Compared with the O atom-end, the C atom-end has more diffuse electron density (as shown in 3σ HOMO, Fig. 1d), which results in negative EP at some distance away (>200 pm) from the C atom-end. Moreover, the electron density around the C nucleus which is more dispersed than that around the O nucleus, causes the region near the C atom perpendicular to the CO axis to be the most electro-positive. It is because the negative EP due to the electron density at a distance from C is exponentially inverse-proportional to the distance, while the positive EP due to the nuclear charge of C/O is inversely proportional to the distance from C/O.

**Figure 2:**
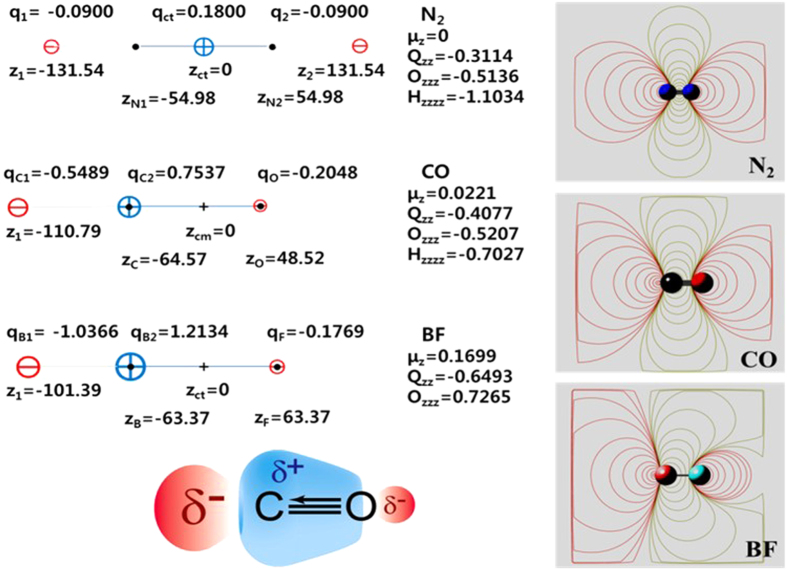
Tripole models (left) which reproduces the EP maps (right) of isoelectronic molecules N_2_, CO and BF at the CCSD(T)/aV5Z level along with a schematic representation (left-bottom) of the EP surface of CO (distances in pm, 2^n^-pole units in e·Å^n^). N_2_ has clearly three divided regions of negative-positive-negative EP in the whole space. CO having a weak dipole is practically three divided regions in the normal range of molecular interaction ranges (within 10^4^ pm), while BF having moderate dipole moment has a small region (within 10^3^ pm) of the negative EP around the F atom-end which is eventually surrounded by positive EP. Thus, BF behaves like a quadrupole in the near F atom-end, but like a dipole in the region far from the F atom-end as if F/B were positively/negatively charged. Here, one can note that the B in BF behaves like a singlet carbene with both positive and negative charges on B respectively along the bond-end and the radial directions of the B atom, while the C in CO partially shows such a behavior. For a comparative study, the tripole models for dihalogen atoms (F_2_, Cl_2_ and Br_2_) are also studied (Supplementary Information: Figure S1). It should be noted that these halogen cases show the opposite EP behaviors to the N_2_, CO and BF cases which give positive EP along the bond-ends but negative EP over the bond-cylindrical surface.

If the NBO charges of C and O were considered isotropic, it could give a very large dipole moment (μ_z_ = ~ −2.4 D) in the opposite direction to the experimental one (0.11 D). Owing to the highly anisotropic behavior in the C/O charge, i.e., electron transfer driven polarization effect, the dipole of CO is very small, and so its EP is strongly quadrupole driven (Q_zz_ = −1.85 D·Å at the coupled cluster with singles, doubles, and perturbative triples (CCSD(T))/aV5Z level; experiment: Q_zz_ = −1.9 ± 0.1/−1.93 ± 0.04 D·Å)^5^,^14^. Hence, CO behaves electrostatically almost like N_2_^15^, opposite to the σ-hole effect of halogen bonding in which the EP of the halogen atom-end is positive^16^,^17^. We note that the EP of CO is well described by the tripole model, as shown in Fig. 2 in which the EPs of isoelectronic molecules CO, N_2_ and BF are compared. The tripole model shows the three divided regions comprised of two negative EP regions around C and O atom-ends and one positive region around the cylindrical surface of the CO bond between C and O. In other words, it can be described as either two dipoles in the opposite direction with slightly different magnitude or the split positive charge of C with a large dipole moment, which results in the dominant quadrupole effect. This tripole model can also be generalized to represent the EP maps of halide dimers showing the σ-hole effect^16^,^17^ (Supplementary Information: Figure S1).

When CO is excited as a triplet state (CO^t^: subscript “t” denotes the first excited state triplet a^3^Π), the triple bond comprised of 1π_x_ 1π_y_, and 3σ bonding orbitals changes to the double bond as one electron in the 3σ bonding orbital excites to the 2π_y_* antibonding orbital (where the x axis is perpendicular to the plane and the y axis is on the plane) (Fig. 1e). Since CO^t^ has now a double bond, the bond distance increases to 120.5 pm (experiment: 120.6 pm)^5^. The overcrowded electron population between the two nuclei in the ground singlet CO is significantly reduced; ergo, the electron population between two nuclei in CO^t^ no longer spills over outside the two nuclei, as in N_2_. The large electron population between the C and O atoms in the σ MO cancels the depleted electron population of the π_y_* MO. The σ_g_ bonding induces the atom-ends to be electrostatically positive along the z axis by overlap between two p_z_ orbitals. Meanwhile, the π_y_* MO induces (i) negative EP due to highly increased electron density on the top and bottom of the cylindrical surface between the two nuclei (Fig. 1b), and induces (ii) positive EP due to the depleted electron density on the front and back of the cylindrical surface (Fig. 1c). The NBO charges of C and O in CO^t^ are + 0.83 and −0.83 au. The dipole moment is −1.39 D (experiment: μ_z_ = −1.374 D)^5^,^18^ and the quadrupole moment is Q_zz_ = 1.41 D·Å. The vertical excitation energy is 6.49 eV (experiment^19^: 6.32 eV).

We note a significant anisotropy in hard wall radius (r_w_) of C and O, specifically, a significant difference between the C/O atom-end directions (C/O-e) and C/O perpendicular directions (C/O-p) (Supplementary Information: Figure S2). At the CCSD(T) complete basis set (CBS)^20^,^21^ limit, the r_w_ (in pm) along the four directions of (C-e, C-p, O-p and O-e) in the singlet CO are (177, 157, 155 and 145), and those in the triplet CO^t^ are (111, 159, 141 and 132). Further, a large difference in r_w_ along the C-e between CO and CO^t^ (177 pm vs. 111 pm) should be noted. The minimum r_w_ for CO (145 pm along O-e) is much larger than that for CO^t^ (111 pm along C-e), which could be utilized for their separation through porous materials.

**Figure 3:**
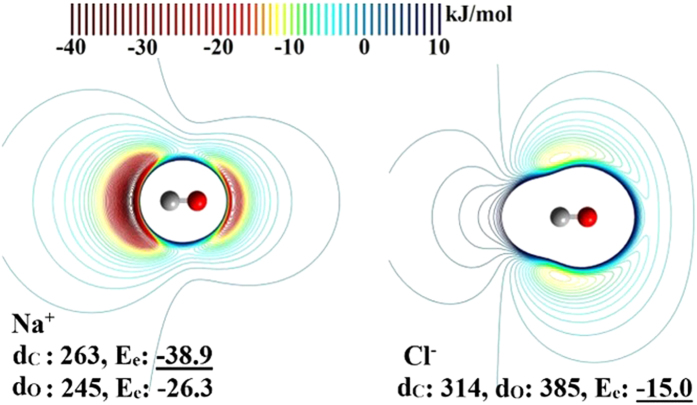
Interactions of CO with Na^+^ and Cl^–^ at the MP2/aVTZ level. E_e_ is in kJ/mol at the CCSD(T)/CBS level; The shortest distance d_C/O_ from C/O to Na^+^/Cl^–^ at the (local) minimum energy potential is given in pm at the CCSD(T)/aVTZ level. Na^+^ has two minima along the C-e and O-e, while Cl^–^ has two identical minima nearly along the C-p.

To better visualize the EP map, the interactions of CO with a cation Na^+^ and an anion Cl^-^ are plotted (Fig. 3). The strongest interaction energy (E_e_) for Na^+^ appears along C-e (E_e_ = −39 kJ/mol), and the second strongest one appears along O-e (E_e_ = −26 kJ/mol) where E_e_ is given at the CCSD(T)/CBS level. On the other hand, Cl^-^ interacts strongly with CO around the cylindrical surfaces of the bond (E_e_ = −15 kJ/mol). This clearly demonstrates that both the C and O atom-ends favor a cation, whereas the cylindrical surface of the CO bond favors an anion with the strongest binding site located slightly nearer to C than O (i.e., along C-p) (Supplementary Information: Figure S2).

**Figure 4:**
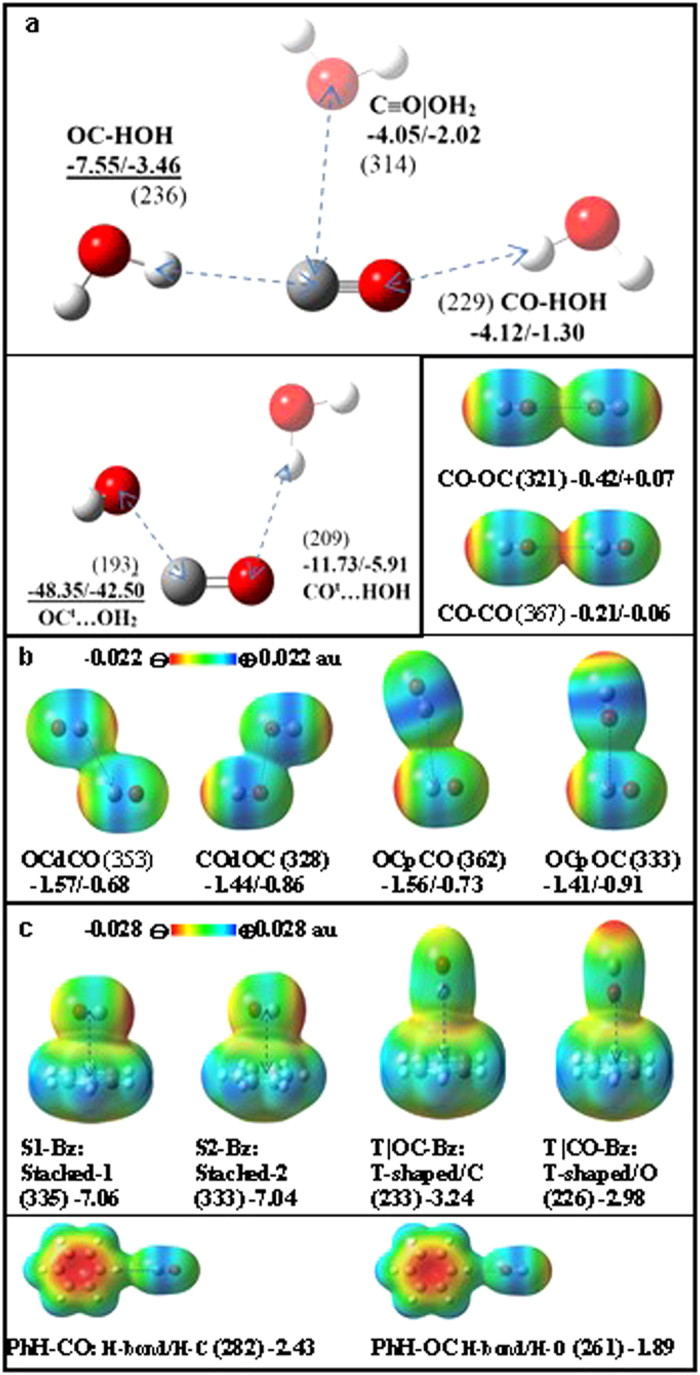
Structures of (a) CO/CO^t^…H_2_O, (b) (CO)_2_ dimer, and (c) CO…Bz. Each distance (pm) marked in a dotted line is given in parentheses at the CCSD(T)/aVTZ optimized geometry. The zero-point-energy (ZPE)-uncorrected/ corrected interaction energies (**E**_**e**_**/E**_**0**_ in kJ/mol in bold) are given at the CCSD(T)/CBS level, using the MP2/aVTZ ZPE correction. EP maps are drawn at the MP2/aVTZ level (density isovalue: 0.001 au).

Now we study the interactions of CO with H_2_O, another CO, and benzene (Bz) to understand their hydrophilic/hydrophobic nature as well as their noncovalent bonding characters (Fig. 4). There have been a few studies on the CO…H_2_O interaction^22^,^23^. In our calculations, the C atom-end moderately interacts with H of H_2_O (E_e_ = −7.55 kJ/mol) at d_C..H_ = 236 pm. The O atom-end weakly interacts with O of H_2_O (E_e_ = −4.12 kJ/mol) at d_O…H_ = 229 pm. The cylindrical surface of the CO bond weakly interacts with O of H_2_O (E_e_ = −4.05 kJ/mol) at d_C…O_ = 314 pm along C-p. It is interesting to note that the C atom interacts with both H and O of H_2_O more favorably than the O atom of CO. Though CO is soluble only in a small amount because of strong water-water interactions, the interaction of H_2_O with CO is not insignificant. The H atoms of H_2_O favorably interact with the C and O atom-ends of negative EP, while the O atom of H_2_O favorably interacts with the cylindrical surface of the CO bond of positive EP.

The binding energies of these CO…H_2_O structures are mainly governed by the electrostatic interaction. Using symmetry adapted perturbation theory (SAPT)^24^,^25^ for quantitative understanding, we perform energy decomposition with the asymptotically corrected PBE0 functional and aVTZ basis set on the MP2/aVTZ optimized geometry. We analyze the SAPT interaction energy components: electrostatic energy (E_es_), effective induction energy (E_ind*_ = E_ind_ + E_exch–ind_), effective dispersion energy (E_disp*_ = E_disp_ + E_exch–disp_), effective exchange repulsion (E_exch*_ = E_exch_ – E_exch-ind_ – E_exch-disp_)^26^,^27^, higher order correction term (E_HF_) and total SAPT interaction energy E_tot_ (Table 1). Since the C-end has is a more negative EP than the O-end in CO, **OC-HOH** (E_es_ = −10.9 kJ/mol) shows stronger electrostatic energy than **CO-HOH** (E_es_ = −4.71 kJ/mol). On this account, the former has much larger binding energy than the latter. **(C≡O)|OH**_**2**_ (E_es_ = −4.87 kJ/mol) also shows weak binding energy because of the positive EP on the large cylindrical surface of the CO triple bond. Meanwhile, CO^t^ interacts more strongly with H_2_O via the OC…OH_2_ electrostatic interaction because of much larger dipole moment than the singlet CO.

In the case of the (CO)_2_ dimer both displaced-stacked (d) and perpendicular (p) structures show similar interaction energies (–E_e_ = 1.6 ~ 1.5 kJ/mol), while the linear structures are hardly bound or not bound mainly due to electrostatic repulsion (**CO-OC**/**CO-CO**: E_es_ = 0.07/0.36 kJ/mol). For most of the (CO)_2_ dimer structures, |E_disp*_| is found to be much larger than |E_es_| (Table 1). However, E_disp*_ tends to be partly cancelled by E_exch*_ at the equilibrium structure. Thus, E_tot_ is close to E_es_ in most cases^28^. Though E_es_ is small, it governs the stability of structures.

In the case of CO…Bz, the stacked conformations of CO on benzene are the most stable (**S1-Bz**/**S2-Bz**: E_e_ = −7.06/−7.04 kJ/mol). They show strong electrostatic energies (E_es_ = −5.63/−5.53kJ/mol), while the effective dispersion and exchange energies nearly cancel each other (E_disp*_ + E_exch*_ = 0.66/0.33 kJ/mol) (Table 1). Thus, the structures are driven electrostatically. Even though the magnitude of the effective dispersion is large (E_disp*_ = −12.52/12.24 kJ/mol), the anisotropic charge distribution in CO governs the structures, because the high density of electron population above benzene showing negative EP is stabilized by the positive EP on the CO cylindrical surface.

The T-shaped structure of CO above benzene where the C is pointing to the benzene centroid is weakly stable (**T|OC-Bz**: E_e_ = −3.24 kJ/mol), while the opposite conformation (the O of CO is pointing to the benzene) is also weakly stable (**T|CO-Bz**: E_e_ = −2.98 kJ/mol), as expected from the similar negative EPs of the C and O atom-ends. The C/O atom-end of CO involves in weak H-bonding with the H atoms of benzene along the benzene side (**PhH-CO**: E_e_ = −2.43 kJ/mol, **PhH-OC**: E_e_ = −1.89 kJ/mol), where the C atom-end has slightly stronger binding energy than the O atom-end due to the C atom-end showing more negative EP than the O atom-end.

Once CO is excited, the highly positively charged C atom-end of CO^t^ favors the negatively charged center of the benzene (**T|OC-Bz**: −17.70 vs. **T|CO-Bz**: −1.75 kJ/mol; **S1-Bz**/**S2-Bz**: −5.56/−16.93 kJ/mol, Supplementary Information: Figure S3). This excitation changes the conformation from the parallel structure of benzene-CO to the perpendicular structure of benzene-CO^t^, which could be exploited as a mechanical device of molecular rotor by alternating laser pulses corresponding to the light absorption and emission between the ground and excited states. Since benzene requires less energy from the singlet to triplet excitation than CO, benzene can be more easily excited to the triplet state than CO. In this case, the CO-Bz^t^ conformational energetics (**S2-Bz**/S**1-Bz**: −7.19/−6.40 kJ/mol; (**T|OC-Bz)**/(**T|CO-Bz)**: −2.60/−2.91 kJ/mol) are somewhat similar to those of CO…Bz (see Supplementary Information: Figures S4 and S5).

## Discussion

In summary, we note that both C and O atom-ends of the CO bond show negative EP (where the C end is slightly more negative), whereas the cylindrical surface of the CO bond axis shows positive EP. This has been properly described by the tripole model which shows the three regions comprised of two negative EP regions around C and O atom-ends and one positive region around the cylindrical surface of the CO bond between C and O. Thus, both C and O atom-ends favor positively charged sites, while the cylindrical surface (in particular near the C atom) favors negatively charged sites. Such phenomena are demonstrated based on the interactions of CO with a cation/anion, H_2_O, and benzene. Clearly CO should not be considered as a molecule with a simple weak dipole moment, but needs to be interpreted as a quadrupole driven molecule (like N_2_) with very weak dipole moment. On the other hand, the triplet CO^t^ has a significant dipole moment; accordingly, the O-end shows negative EP, while the C-end shows positive EP. The present results could further facilitate diverse gas phase experiments involving CO-bound complexes.

## Methods

EP maps and most of the MP2 calculations were carried out using the GAUSSIAN09 suite of programs^24^. Molecular orbitals were investigated at the M06-2X level of theory^29^ using aug-cc-pVTZ basis set. Most of the CCSD(T) calculations were performed using the MOLPRO software^30^. SAPT calculations were carried out using SAPT2012^32^.

The optimized geometry (z_C_ = 64.57 pm and z_O_ = 48.52 pm) and multipole moments of CO were calculated at the CCSD(T)/aug-cc-pV5Z level. Since the analytic derivatives to calculate multipole moments are not available for the CCSD(T) method, numerical differentiation using the field strength of 0.0005 au was carried out. The perturbation Hamiltonian H’ of the following form is added to the one-electron Hamiltonian, and the corresponding CCSD(T) energy E(λ) is computed.


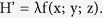


Then, the corresponding moment is approximated using the four-point formula:


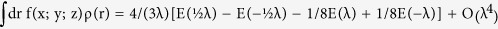


For the calculation of physically meaningful moments, perturbation Hamiltonians are designed according to the definitions for the multipole moments given below.


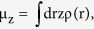



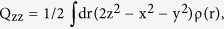



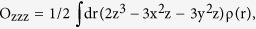



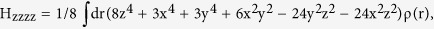


where ρ(r) includes both nuclear charge and electron density.

With the accurate multipole moments at hand, we attempt to design a system composed of three point charges q_C1_, q_C2_ and q_O_ placed at z_C1_, z_C2_; and z_O_ (q: charge, z: coordinate along the CO bond axis) which reproduces the calculated moments including the monopole moment (charge balance).

The geometry and multipole moments of CO were obtained at the CCSD(T)/aug-cc-pV5Z level. The three point charges are given by (q_C1_ = −0.5843 au, z_1_ = −109.48 pm), (q_C2_ = + 0.7917 au, z_2_ = −65.43 pm), and (q_O_ = −0.2074 au, z_O_ = + 47.75 pm), while the atomic sites are located at −64.57 pm (C) and + 48.52 pm (O), where the center of mass is at the origin. This model represents that a negative charge (−0.5843 au) is at the distance of 44.91 pm along the C atom-end from the C atom, a large positive charge (+0.7917 au) is at the distance of 0.86 pm from the C atom toward the O atom, i.e. very close to the C atom, and a small negative charge (−0.2074 au) is at the distance of 0.77 pm from the O atom site. This indicates that a large negative charge is at the distance 45 pm from the C atom-end and a small negative charge is near the O atom-end, while a positive charge is near the C atom. This electrostatic potential can be nearly exactly represented as a more simplistic tripole model in which the actual negative charges are at a certain distance from the C atom-end and at the position of the O atom, and the positive charge is at the C atom.

The simplified three site model has (q_C1_ = −0.5489 au, z_1_ = −110.79 pm), (q_C2_ = + 0.7537 au, z_2_ = −64.57 pm, i.e. the C nucleus site), and (q_O_ = −0.2231 au, z_O_ = + 48.52 pm, i.e., the O nucleus site), as shown in Fig. 2, without any significant difference in electrostatic potential from the previous model. Thus, this simplified model is finally chosen as the electrostatic potential of CO. This indicates that a large negative charge protrudes from the C nucleus (at the distance of 46.22 pm from the C nucleus), and a small negative charge is at the O nucleus site, while a large positive charge is at the C nucleus site, as schematically shown in Fig. 2. We further compared isoelectric molecules N_2_, CO and BF in Fig. 2. Finally, the N_2_, CO and BF cases are compared with the dihalogen cases of F_2_, Cl_2_ and Br_2_ which show positive EP along the bond-ends but negative EP over the bond-cylindrical surface (i.e., opposite EP behaviors to the N_2_, CO and BF cases; Supplementary Information: Figure S1).

## Additional Information

**How to cite this article**: Kim, H. *et al.* Intriguing Electrostatic Potential of CO: Negative Bond-ends and Positive Bond-cylindrical-surface. *Sci. Rep.* **5**, 16307; doi: 10.1038/srep16307 (2015).

## Supplementary Material

Supplementary Information

## Figures and Tables

**Table 1:** SAPT-DFT energy decomposition (kJ/mol) of CO…H_2_O, (CO)_2_, and CO…Bz with the asymptotically corrected PBE0 functional and the aVTZ basis set on the MP2/aVTZ optimized geometries.

Complex	E_es_	E_ind_^*^	E_disp_^*^	E_exch_^*^	δH_HF_	E_tot_	E_CCSD(T)/CBS_
OC-HOH	−10.90	−2.22	−5.80	13.13	−1.39	−7.18	−7.55
CO-HOH	−4.71	−1.09	−3.95	6.51	−0.52	−3.75	−4.12
C≡O|OH_2_	−4.87	−0.72	−4.11	6.04	−0.31	−3.96	−4.05
OCdCO	−1.49	−0.10	−2.55	2.79	−0.22	−1.57	−1.57
COdOC	−0.97	−0.02	−2.59	2.34	−0.08	−1.31	−1.44
OCpCO	−1.47	−0.09	−2.39	2.54	−0.13	−1.54	−1.56
OCpOC	−1.10	−0.05	−2.48	2.44	−0.10	−1.29	−1.21
CO-OC	0.07	−0.02	−1.84	1.53	−0.04	−0.30	−0.42
CO-CO	0.36	−0.03	−1.34	1.00	−0.04	−0.05	−0.21
S1-Bz	−5.63	−0.43	−12.52	13.18	−1.51	−6.91	−7.06
S2-Bz	−5.53	−0.42	−12.24	12.57	−1.43	−6.95	−7.04
T|OC-Bz	−1.71	−0.38	−10.94	10.93	−1.24	−3.34	−3.24
T|CO-Bz	−1.01	−0.03	−8.47	7.83	−0.65	−2.44	−2.98
PhH-CO	−2.80	−0.26	−3.19	5.07	−0.35	−2.26	−2.43
PhH-OC	−1.84	−0.11	−3.48	4.08	−0.24	−1.58	−1.89
