# Supplementary material for: Intriguing Electrostatic Potential of CO: Negative Bond-ends and Positive Bond-cylindrical-surface
Source: Sci Rep. 2015 Nov 6;5:16307. doi: 10.1038/srep16307 (PMC4635358; doi:10.1038/srep16307)
Supplement: Supplementary Information [file srep16307-s1.pdf]

Supplementary Information for:

Intriguing electrostatic potential of CO: negative bond-ends and positive bond-cylindrical-surface

Hahn Kim[1], Van Dung Doan[2], Woo Jong Cho[2], Rosendo Valero[2], Zahra Aliakbar Tehrani[2], Jenica Marie L. Madridejos[2] and Kwang S. Kim[2*]

[1]Department of Chemistry, Korea Advanced Institute of Science and Technology, Daejeon 305-701, Korea

[2]Department of Chemistry, School of Natural Science, Ulsan National Institute of Science and Technology (UNIST), Ulsan 689-798, Korea

*Correspondence and requests for materials should be addressed to K.S.K. (email: kimks@unist.ac.kr)

Supplementary Figures

Supplementary Figure S1 | Generalization of the tripole models for the EPs of dihalogen molecules (F_2, Cl_2 and Br_2) (distances in pm; 2^n-pole moments in e·Å^n).

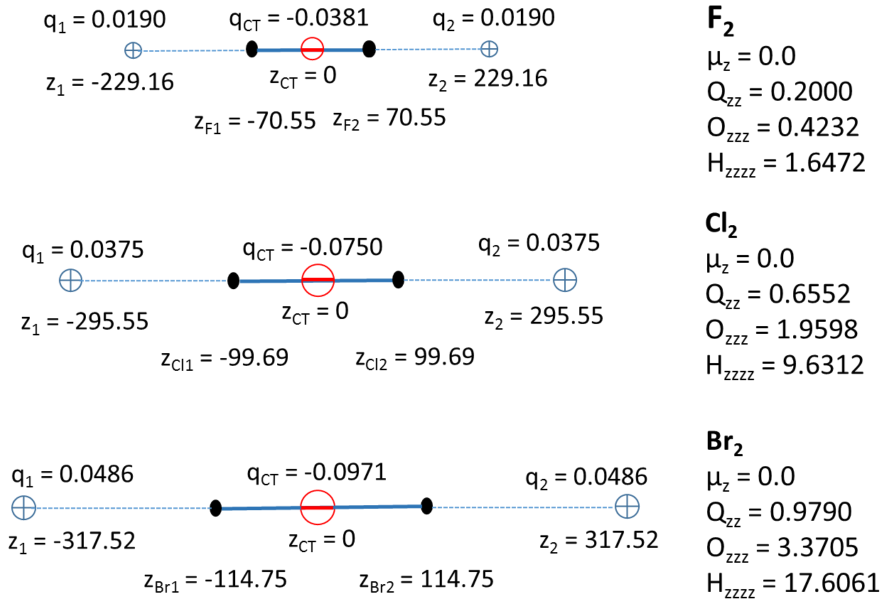

Supplementary Figure S2 | van der Waals radius of CO and CO^t

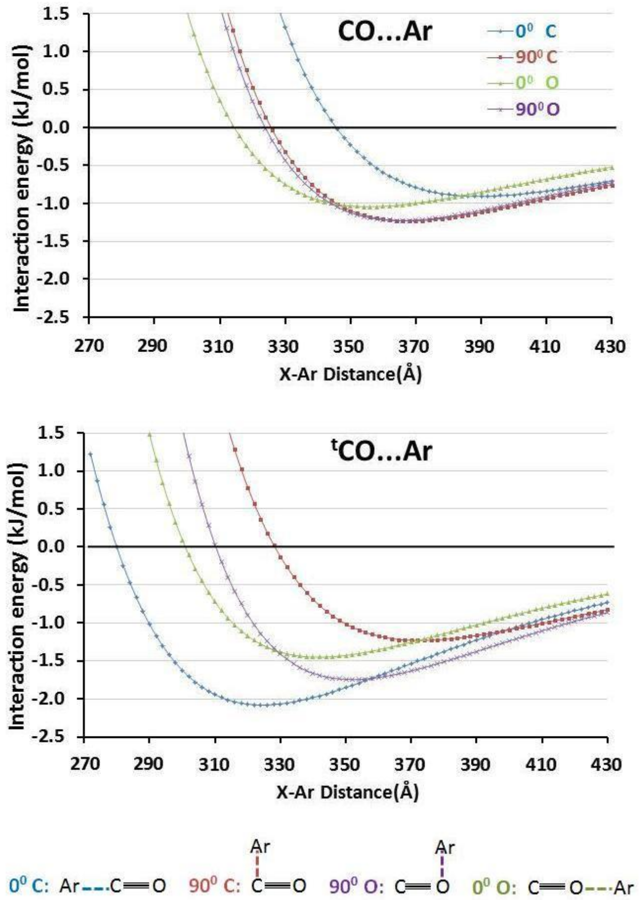

Anisotropic hard wall distance r_w and van der Waals radius r_{vdw} (pm) of an atom C/O in singlet CO and triplet CO^t for four different orientations (0^0C, 90^0C, 90^0O, 0^0O) at the CCSD(T)/CBS level. r_w: CO (346, 326, 324, 314) and CO^t (280, 328, 310, 301). r_v: CO (388, 368, 366, 356) and CO^t (324, 373, 352, 342) at the CCSD(T)/CBS level. The van der Waals interactions composed of dispersion energies (E_{disp}) and exchange repulsion energies (E_{exch}) determine the molecular size. The van der Waals radii of atoms are generally treated isotropically because their features are considered to be hardly susceptible to the environmental effects. Nevertheless, we note a significant anisotropy in van der Waals radius (r_v) of C and O, namely, a significant difference between the $\theta=0^o$ direction (atom-ends) and the $\theta=90^o$ direction (Figure S2). By excluding the hard wall radius (r_w) of Ar (r_w=169 pm; r_v=188 pm), the r_w values (in Å) for the cases (C:$\theta=0^o$/C:$\theta=90^o$/O:$\theta=90^o$/O:$\theta=0^o$) in the singlet CO are (177, 157, 155, 145), and those in the triplet CO^t (111, 159, 141, 132) at the CCSD(T) complete basis set (CBS) limit value (based on the extrapolation scheme using the single-point CCSD(T)/aVTZ and CCSD(T)/aVQZ energies on the CCSD(T)/aVTZ optimized geometry). Their van der Waals radii are similar to but slightly larger than 1.122(=$2^{1/6}$) times that of the r_w: r_v: CO (200, 180, 178, 168) and CO^t (136, 185, 164, 154). A drastic change in r_w along the C atom-end (0^0C) between CO and CO^t (200 pm vs. 136 pm) should be noted.

Supplementary Figure S3 | Structures of benzene(singlet)...CO(triplet)

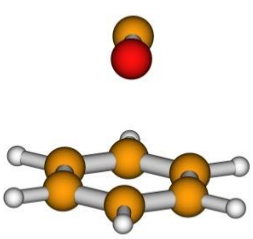

S1-Bz
(277) **-15.56**

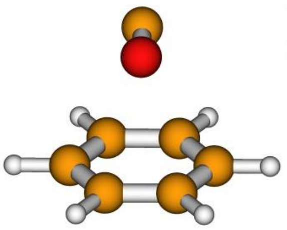

S2-Bz
(264) **-16.93**

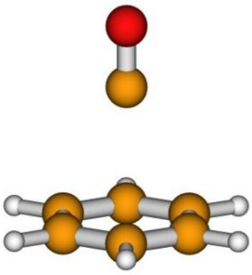

T|OC^t-Bz
(271) **-17.70**

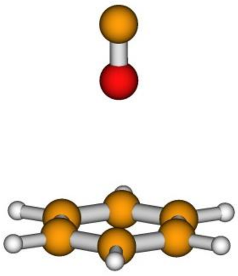

T|CO^t-Bz
(317) **-1.75**

The CCSD(T)/aVTZ interaction distances are given in pm in parentheses.
The CCSD(T)/CBS interaction energies are given in kJ/mol in bold characters.

Supplementary Figure S4 | Geometry for the triplet benzene

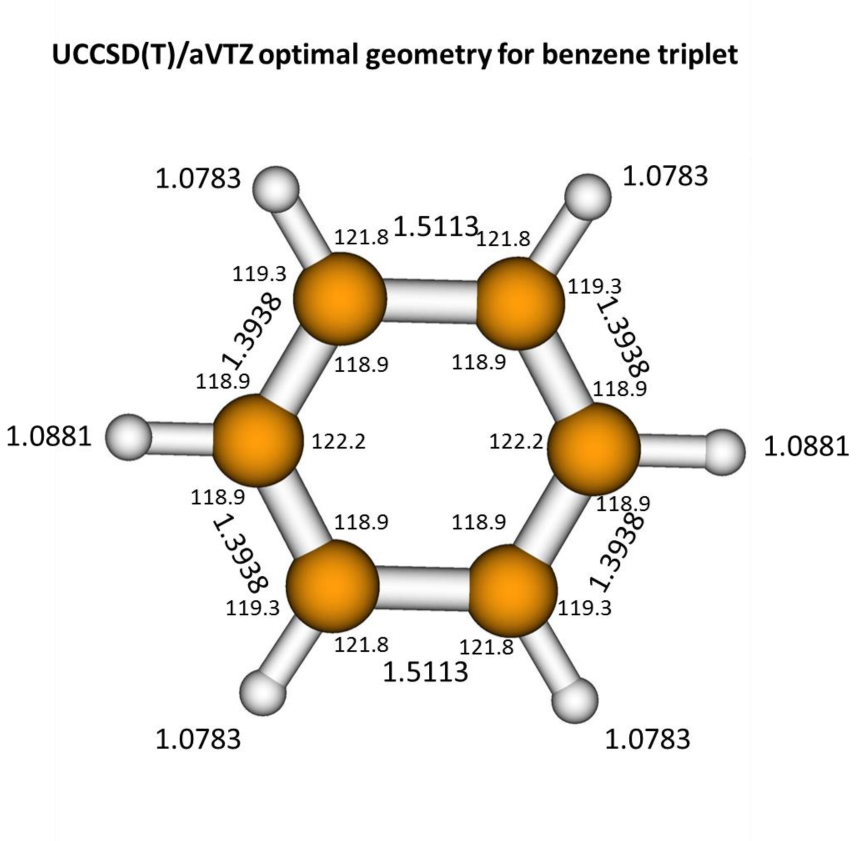

Supplementary Figure S5 | Structures of benzene(triplet)...CO(singlet)

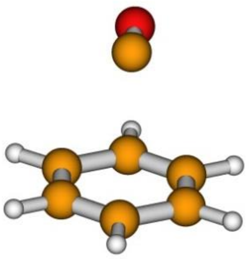

S1-Bz
(334) **-6.45**

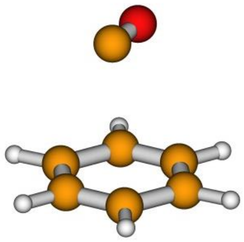

S2-Bz
(336) **-6.16**

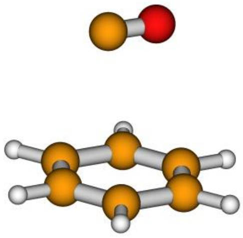

S3-Bz
(332) **-6.40**

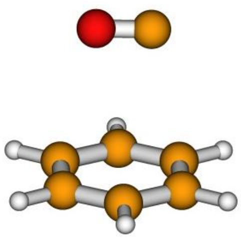

S4-Bz
(327) **-7.19**

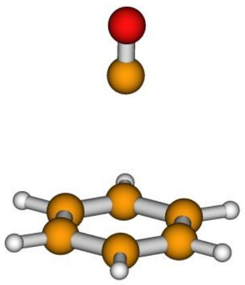

T|OC-Bz
(342) **-2.60**

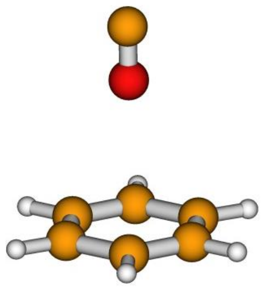

T|CO-Bz
(315) **-2.91**

The CCSD(T)/aVTZ interaction distances are given in pm in parentheses.
The CCSD(T)/CBS interaction energies are given in kJ/mol in bold characters.
Two longer C-C bond distances are shown to be the shortest in the triplet benzene.
